# Neural network-based algorithm for door handle recognition using RGBD cameras

**DOI:** 10.1038/s41598-024-66864-7

**Published:** 2024-07-09

**Authors:** Lesia Mochurad, Yaroslav Hladun

**Affiliations:** Lviv Polytechnic National University, Lviv, 79013 Ukraine

**Keywords:** Robotics, RGBD camera, Neural network, Framework TensorFlow, The Mean Squared Error, Engineering, Mathematics and computing

## Abstract

The ability to recognize and interact with a variety of doorknob designs is an important component on the path to true robot adaptability, allowing robotic systems to effectively interact with a variety of environments and objects The problem addressed in this paper is to develop and implement a method for recognizing the position of a door handle by a robot using data from an RGBD camera. To achieve this goal, we propose a revolutionary approach designed for autonomous robots that allows them to identify and manipulate door handles in different environments using data obtained from RGBD cameras. This was achieved by creating and annotating a complete dataset consisting of 5000 images of door handles from different angles, with the coordinates of the vertices of the bounding rectangles labeled. The architectural basis of the proposed approach is based on MobileNetV2, combined with a special decoder that optimally increases the resolution to 448 pixels. A new activation function specially designed for this neural network is implemented to ensure increased accuracy and efficiency of raw data processing. The most important achievement of this study is the model's ability to work in real-time, processing up to 16 images per second. This research paves the way for new advancements in the fields of robotics and computer vision, making a substantial contribution to the practical deployment of autonomous robots in a myriad of life's spheres.

## Introduction

An autonomous mobile robot represents a sophisticated, self-operating mechanism capable of independent functioning and navigation within its designated environment, without the need for constant human oversight or intervention. Utilizing an array of sensors^1,2^, these robots perceive their surroundings, process acquired data, and make decisions to accomplish specific tasks. They integrate advanced technologies such as artificial intelligence, computer vision, machine learning, and route planning algorithms to effectively perceive, interpret, and interact with their environment. Autonomous mobile robots have diverse applications across logistics, manufacturing, healthcare, agriculture, research, and transportation, where they autonomously execute tasks, thereby enhancing productivity and safety. Additionally, they play significant roles in service and maintenance tasks, alleviating burdens in daily human life by undertaking household chores, assisting individuals with disabilities, and providing companionship. Their ability to continually acquire new skills and adapt to changing environmental conditions makes autonomous robots invaluable assistants across various facets of contemporary existence.

The recognition and manipulation of door handles constitute a crucial component in the realm of contemporary robotics and automation, asserting a significant impact across a multitude of domains. For autonomous robotic systems, such as service or delivery robots, proficiency in indoor handling is imperative^3,4^, as it facilitates seamless transit between different spaces, thereby enabling autonomous navigation in multifaceted environments.

Despite the advancements in door handle recognition and manipulation by autonomous robots, significant gaps remain in the literature. Most existing methods are limited by their inability to operate effectively in real-time or under varying lighting conditions. For instance, many state-of-the-art algorithms exhibit reduced accuracy and efficiency when confronted with changes in illumination, which is a common occurrence in practical deployment scenarios. Additionally, the computational demands of these methods often prohibit real-time application, rendering them impractical for dynamic environments where rapid decision-making is crucial.

Another notable gap is the adaptability of these methods to different types of door handles and environments. Current solutions frequently rely on extensive pre-programming or specific training datasets that do not generalize well across different settings. This lack of versatility hampers the widespread adoption of autonomous robots in real-world applications, such as healthcare facilities, industrial settings, and home automation systems, where the variety of door handle designs and environmental conditions is vast.

Furthermore, the integration of depth information with RGB data, while promising, has not been fully exploited in many existing approaches. Most methods predominantly focus on either RGB or depth data, failing to harness the combined potential of RGBD cameras to enhance accuracy and robustness. This oversight results in suboptimal performance, particularly in complex scenarios requiring precise spatial awareness.

This research aims to fill these gaps by developing a robust and efficient method for door handle recognition using an RGBD camera, capable of real-time operation. By leveraging the full spectrum of data provided by RGBD cameras, our approach enhances the robot's ability to accurately detect and interact with door handles under diverse lighting conditions and in various environments. Additionally, the proposed method integrates advanced machine learning techniques to ensure rapid processing and decision-making, enabling real-time application. Through comprehensive testing and validation, we demonstrate the method's superiority in both accuracy and operational efficiency, paving the way for more versatile and reliable autonomous robotic systems.

The primary objective of this work is the development and optimization of a method for the recognition of door handle positions utilizing data from an RGBD camera. This encompasses the exploration and implementation of computer vision and machine learning methodologies, such as Convolutional Neural Network (CNN), alongside the creation of specialized algorithms for the analysis of received data and the ascertainment of the door handle's isometry.

In the sphere of safety and assistance, robots endowed with the capability to autonomously operate doors play a pivotal role, particularly in aiding individuals with disabilities or the elderly^5^. This function not only enhances their mobility within residential settings but also promotes a greater degree of independence. In the context of search and rescue operations^6^, the robotic ability to manipulate doors becomes critically valuable, especially in scenarios of emergencies such as fires or earthquakes. Robots, capable of accessing perilous zones, can potentially save lives by conducting search and rescue missions in situations where human intervention carries substantial risk.

Within the ambit of intelligent home and office systems^7^, robotic interaction with door handles can be leveraged to manage access and bolster security, thereby augmenting the comfort and efficiency associated with the use of such intelligent systems. In industrial environments, robots capable of transporting materials across various sections, segregated by doors, contribute to a more flexible and efficient production process^8^. The automation of door operation paves the way for the development of more integrated production lines.

Moreover, in the field of research and exploration, the ability of robots to open doors allows for the investigation of extreme environments, such as space missions or underwater explorations^9–11^, significantly reducing risks to human life. This capability is instrumental in extending the frontiers of human knowledge, as it enables robotic exploration in conditions that are otherwise inaccessible or hazardous for human beings.

The real-time operational capability of the model heralds significant prospects across various sectors where the celerity of response is of paramount importance^12,13^. In the realms of robotics, autonomous vehicular systems, and video surveillance frameworks, the immediacy in responding to incoming data can avert potential incidents necessitating urgent intervention. This empowers autonomous systems to rapidly formulate decisions, adeptly adapting to alterations in their ambient environment.

The motivation for this paper stems from the key role that autonomous mobile robots play in various fields, including logistics, manufacturing, healthcare, and transportation, where their ability to perform tasks autonomously increases productivity and safety. In particular, recognizing and manipulating door handles is a critical task in modern robotics that has significant implications for various sectors. For example, in the area of security and assistance, autonomous robots capable of manipulating doors can significantly help people with disabilities or the elderly by facilitating their mobility and independence. In addition, in industrial plants, the interaction of robots with door handles contributes to more flexible and efficient production processes. In addition, the ability of robots to open doors is crucial for research and exploration in extreme environments, such as space missions or underwater exploration, where human access is limited.

The primary objective of this work is the development and optimization of a method for the recognition of door handle positions utilizing data from an RGBD camera. This encompasses the exploration and implementation of computer vision and machine learning methodologies, such as Convolutional Neural Network (CNN), alongside the creation of specialized algorithms for the analysis of received data and the ascertainment of the door handle's isometry.

The principal contributions of this article are summarized as follows:

1. An investigation and analysis of existing object recognition and image segmentation methodologies.
2. The proposition of a model architecture based on MobileNetV2, integrated with a bespoke decoder for optimal resolution enhancement.
3. The development of an innovative approach enabling autonomous robots to identify and interact with door handles in diverse environments, based on real-time RGBD data.
4. The execution of verification and validation procedures for the proposed algorithm on an array of door handles under varying lighting conditions and backgrounds, coupled with an analysis of the accuracy and reliability of the developed recognition system to ascertain its practical applicability in real-world robotic scenarios.

The structure of the remainder of the article is as follows: “State-of-the-arts” section provides an analysis of different approaches to door opening by autonomous robots and an overview of existing analogaazs in controlling mobile robotic manipulators. In “Task” section articulates the research problem. The proposed algorithm is detailed in “Proposed algorithm” section. In “Results of numerical experiments” section elaborates on the test data and the results obtained. Finally, the conclusions and prospects for future research are presented in the “Conclusions” section.

## State-of-the-arts

Existing research on door handle recognition and manipulation by autonomous robots can be broadly categorized into three main approaches: methods utilizing RGB cameras for image recognition, techniques employing depth sensors for spatial analysis, and hybrid approaches integrating both RGB and depth data. Within these categories, various algorithms have been developed, including YOLOv5 for object detection, convolutional neural networks for feature extraction, and reinforcement learning for control optimization.

In their scholarly article^14^, Wang et al. elucidate a methodology designed to enhance the reliability of controlling a mobile robotic manipulator in scenarios characterized by non-deterministic environmental behaviors. The method incorporates an augmented Proximal Policy Optimization (PPO) algorithm. The focal point of this research is the application of mobile robotic arms for door opening. The process commences with the identification of the door handle's position using an image recognition technique predicated on YOLOv5^15,16^. Subsequently, the interaction between the robotic arm and its environment is simulated utilizing the CoppeliaSim platform^17^. Following this, a control strategy founded on a reward function is formulated to facilitate the training of a robotic arm to open doors within actual environmental settings. Experimental outcomes demonstrate that this proposed methodology expedites the convergence of the learning process and notably diminishes minor movements of the robotic arm, thereby augmenting control stability.

In their research endeavor^18^, Arduengo et al. have developed a framework tailored for the robust and adaptive operation of conventional doors by an autonomous mobile manipulator. This framework merges a convolutional neural network with efficient point cloud analysis to enhance both robustness and speed performance. This innovative approach allows for the real-time ascertainment of door handle grasping postures using RGBD imagery, a crucial aspect of human-centered assistive applications. Additionally, a multifaceted Bayesian framework is proposed, enabling the robot to analyze the door's kinematic model based on motion observations and incorporate prior experiences or learning derived from human demonstrations. The amalgamation of these algorithms with the Task Space Region motion planner^19^ facilitates proficient interaction with doors, irrespective of their specific kinematic models. The efficacy of this framework has been empirically validated through practical experiments conducted with the Toyota Human Support Robot^20^.

In their scholarly work^21^, Stuede et al. introduce a strategy for door opening employing the KUICA KMR iiwa mobile robot^22^. This robot demonstrates autonomous capabilities in recognizing doorknobs, opening doors, and navigating through doorways without pre-existing knowledge of the door models or geometries. A CNN architecture is utilized for doorknob detection, offering robustness across various doorknob designs and colors. A 100% success rate in detection was achieved among a diverse set of 38 door handles, utilizing the highest confidence level as the selection criterion. The door's plane was identified through the segmentation of depth data, facilitating the creation of bounding boxes for the handles. This methodology operates in real-time on an external computer, achieving approximately 20 Hz, constrained by limited access to the KMR iiwa's internal control loops. Employing this approach, the robot successfully opened doors and navigated through them in 22 out of 25 trials across five distinct doors.

In their scholarly publication, Quintana et al.^23^ introduce a novel methodology for the detection of open and closed doors within 3D laser scanning data, a pivotal task for the indoor navigation of autonomous mobile robots. Their approach synthesizes both geometric (XYZ coordinates) and chromatic (RGB/HSV) data, derived from a calibrated 3D laser scanner coupled with a color camera. The method is developed within the ambit of a sophisticated 6D spatial structure, integrating geometric and color features. This integration, along with other distinctive attributes of the methodology, imparts robustness against overlaps and the capacity to accommodate minor door color variations, which may arise due to disparate lighting conditions encountered during scanning at various locations. The efficacy of this method has been validated through assessments conducted on both simulated and real-world scenarios, yielding promising outcomes.

Additionally, the approach proposed by Mochurad et al.^24^ for door handle detection warrants attention. Their article delineates a novel algorithm that empowers autonomous mobile robots to operate a range of door types independently, without human intervention. This algorithm employs machine learning techniques, notably the YOLOv5 object detection model, the iterative RANSAC method for parameter estimation, and the DBSCAN clustering algorithm^25^, with comparative analyses of alternative clustering techniques also undertaken. The algorithm's performance was evaluated in both simulated and real-world contexts, achieving a notable success rate of 95% in 100 door-opening attempts. The doorknob detection algorithm exhibited an average localization error of 1.98 mm across 10,000 samples, underscoring its precision in pinpointing actual doorknobs. These results attest to the algorithm's accuracy and its efficacy for real-time, autonomous operation across diverse door types. Nonetheless, the experiments identified certain limitations of this approach. The method relies on selecting the optimal cluster and ascertaining its centroid as the door handle's position, necessitating a specific angle between the camera and the door handle. Deviations from this requisite angle may lead to centroid displacement, potentially culminating in unsuccessful door operation attempts. Moreover, consistently achieving a favorable joint configuration of the robot arm that ensures the desired angle between the camera and the door plane is not invariably feasible.

Unlike previous methods, the proposed MobileIsometryDet model mitigates the high processing time and limited generalization issues by leveraging the efficient MobileNetV2 architecture combined with a bespoke decoder. This innovative architecture enables the model to perform real-time operation and deliver robust performance across diverse lighting conditions and door handle designs. By utilizing an optimized decoder, the MobileIsometryDet model significantly enhances the resolution and accuracy of door handle detection, ensuring that the system can function effectively in varied and dynamic environments. Additionally, the integration of a specialized activation function tailored for this architecture improves the overall efficiency of raw data processing, further contributing to the model's high accuracy and reliability. These advancements position the MobileIsometryDet model as a superior solution for real-world applications where speed, accuracy, and adaptability are critical.

Table 1 in the publication concisely enumerates the advantages and disadvantages of each of the existing analogous methods under review, providing a comprehensive comparative analysis.

**Table 1:** Overview of existing analogues.

References	Sensor	Method	Advantages	Disadvantages
Wang et al*.*^14^	RGB camera	YOLOV5 is applied to door-handle recognition. Trust region policy optimization. Proximal policy optimization is used in robotic arm control	Lots of useful information about the environment	Expansive and height processing time
Arduengo et al*.*^18^	6-axis force sensor, located on the wrist, and an RGBD camera, located on its head	Labeling scanned points as either ground or non-ground	Lots of useful information about the environment	Expansive and height processing time
Stuede et al*.*^21^	RGBD camera	Divide into either road ground or obstacles based on the average height of each line segment	A method to effectively deal with obstacles of different heights on city roads	Some limitations in distinguishing dynamic obstacles such as pedestrians
Quintana et al*.*^23^	3D LiDAR and RGB camera	Quantized digital elevation map and grayscale reconstruction	Data processing by using existing image processing techniques	Not discuss the conversion between different scene
Mochurad et al.^24^	Intel Realsense D435 RGBD camera	Finding the door plane using RANSAC. Clustering with DBSCAN. Finding a bounding box that has a door handle using YOLOv5	Exhibits strong reliability when both the door handle and the door meet the specified criteria	Requires a specific angle between the camera and the door handle / door. Works poorly with non-standard door handles

Therefore, various approaches have been analyzed above, including the use of convolutional neural networks, optimization algorithms, image recognition techniques, interactive simulations, and the integration of these technologies to perform specific tasks such as opening doors.

## Task

### Input data for model training

**Figure 1:**
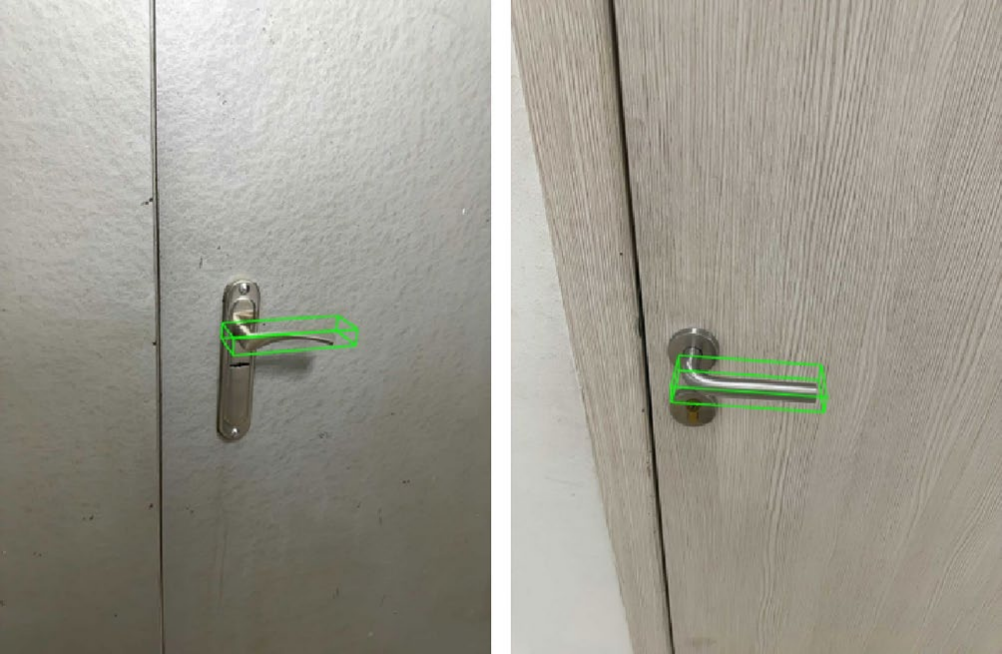
Visualization of two doors from the dataset and their labels in the form of a green BB.

To facilitate the training of the model, a meticulously annotated dataset was curated, comprising 5000 images. Each image is accompanied by the corresponding coordinates of the bounding box (BB) vertices of the door handle, situated within the camera's coordinate framework. The dataset's labeling is visually represented in Fig. 1, where two doors from the dataset are displayed, accompanied by their respective labels, indicated by a green BB.

In a formal capacity, the dataset is represented as an ordered pair of tensors, denoted as $\left( {X_{data} , y_{data} } \right)$ where $X_{data} \in {\mathbb{R}}^{n \times h \times w \times 3}$ corresponds to the tensor of images and $y_{data} \in {\mathbb{R}}^{n \times 24}$ corresponds to the tensor of BB coordinates. Here, $n$ denotes the total number of data points within the dataset, amounting to 5000, $h$ represents the height of the images, and $w$ denotes the width of the images. It is pertinent to note that the dataset utilized for this research endeavor is not available for public access.

### Input data of the final algorithm

**Figure 2:**
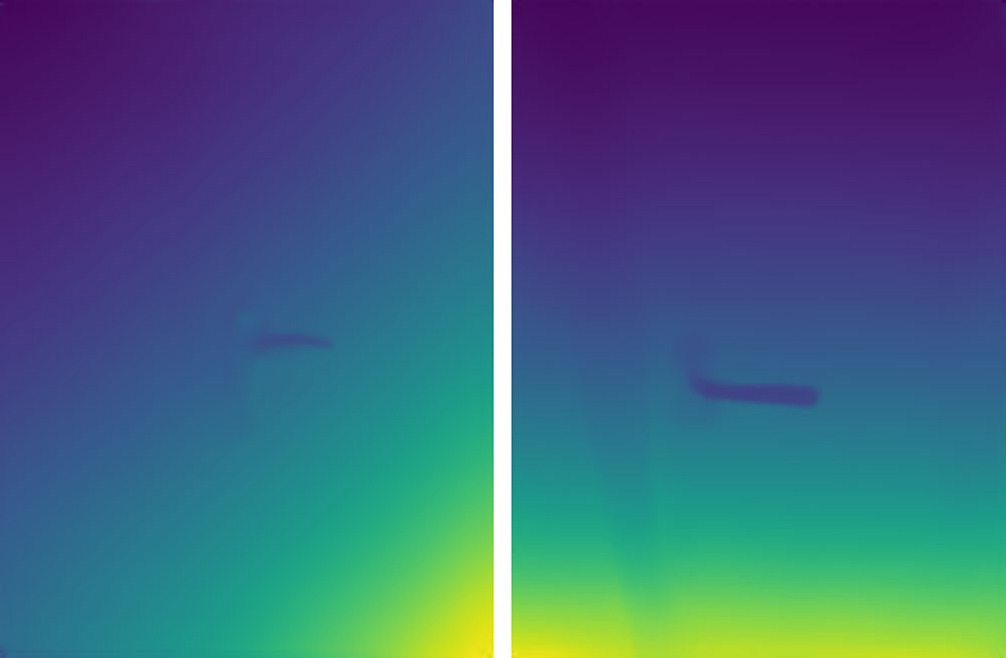
Visualization of the corresponding depth pictures to the data from the dataset, which are previously presented in Fig. 1.

The present work is primarily concentrated on the formulation and refinement of a neural network designed for the identification of bounding box (BB) coordinates, in conjunction with a specialized algorithm for ascertaining isometry. This process utilizes depth imagery and the coordinates of the vertices of the BB. The principal inputs requisite for the algorithm encompass:

1. An RGBD image, as exemplified in Fig. 2 of the publication;
2. The intrinsic parameters of the camera, which are instrumental in translating the depth information of the image into a three-dimensional point set.

These data inputs are critically analyzed and processed by the algorithm to ascertain with precision the spatial position and orientation of the door handles. This process involves meticulous computations and the application of advanced image-processing techniques to ensure accurate results. The integration of these components is pivotal in the algorithm's ability to reliably determine the exact spatial parameters necessary for effective interaction with door handles in varying environmental settings.

There are no stringent specifications regarding the orientation of the camera relative to the door handle in the dataset. It is imperative that the data utilized during the model's operation closely resemble the training data in terms of visual characteristics and perspective. Optimally, the identical camera, equipped with the same intrinsic parameters, should be employed throughout both the training and application phases. This ensures that the camera's viewing angles on the door handle during model deployment closely approximate those captured in the dataset.

### Output data of the final algorithm

The output data of the final algorithm can be considered a pair of vectors $\left( {p^{*} , q^{*} } \right)$, where $p^{*} \in {\mathbb{R}}^{3}$ is a vector responsible for position, $q^{*} \in {\mathbb{R}}^{4}$ is a quaternion responsible for orientation. $p^{*}$ and $q^{*}$ specify the position and orientation of the middle of the door handle.

## Proposed algorithm

MobileNetV2^26^, a significant advancement in the sphere of deep learning and computer vision, was developed by the Google AI team. This model, evolving from its predecessor MobileNet, is architecturally optimized for heightened efficiency, a crucial attribute for deployment in mobile devices and systems with constrained computational resources. Distinguished for its high accuracy, MobileNetV2 achieves this with considerably fewer parameters in comparison to larger, more intricate models. A hallmark of MobileNetV2 is the incorporation of inverted residuals and linear blocks, innovations that underpin the model's efficiency. This structural design enables the reduction of computational demand and the number of parameters, without compromising recognition quality. Such characteristics render MobileNetV2 particularly suitable for real-time applications where swift data processing is imperative. Employed extensively in various computer vision tasks like image classification, object detection, and segmentation, MobileNetV2's versatility and efficiency have led to its widespread application in mobile applications, robotics, automated video surveillance systems, and other areas requiring fast and reliable image processing within the bounds of limited computational resources. Its adeptness in handling diverse data types solidifies its role as a vital tool in the contemporary realm of artificial intelligence. The MobileNetV2 framework represents an evolutionary advancement of the prototypical MobileNet structure, crafted with an emphasis on augmented operational efficiency for deployment across mobile apparatuses and devices characterized by computational resource constraints. Salient Features of MobileNetV2:

Inverted Residuals: This pivotal innovation underpins MobileNetV2's architecture, facilitating the propagation of features via "inverted residuals" with minimal informational attrition.
2. Linear Bottlenecks: Positioned at the terminal phase of each block, these bottlenecks are instrumental in preserving the representational integrity of features.

The dimensionality of the MobileNetV2 model, or the weight file, is contingent upon the selected configuration and the applied depth multiplier. MobileNetV2 proffers an array of models varying in “depth multipliers” and input resolutions, which confer the flexibility to calibrate the equilibrium between precision and model dimensions.

For instance, the archetypal iteration of MobileNetV2, utilizing a depth multiplier of 1.0 and an input dimension of 224 × 224 pixels, typically encompasses a model size approximating 14 megabytes (MB). Conversely, opting for a reduced depth multiplier, such as 0.5, can result in a commensurately reduced model size.

### Proposed architecture MobileIsometryDet

**Figure 3:**
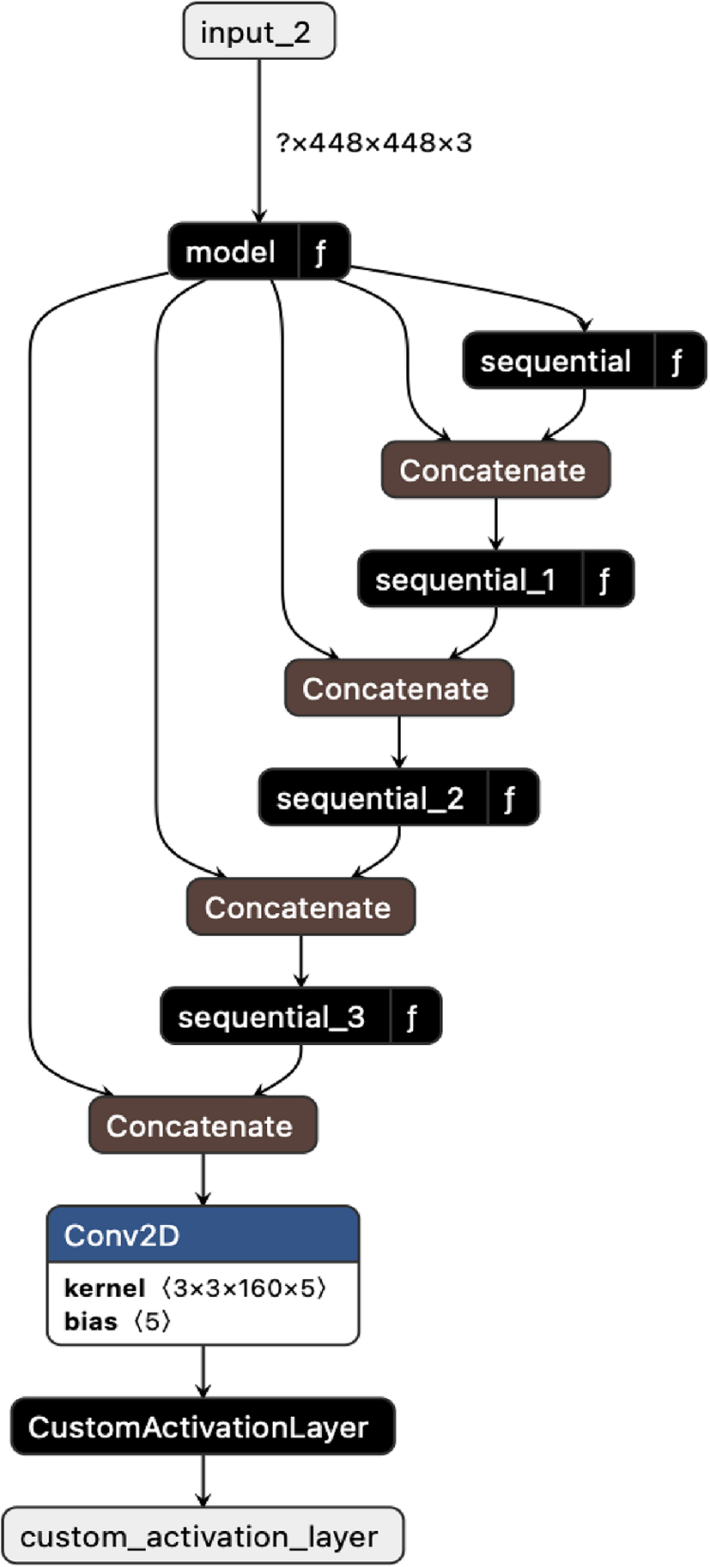
Scheme of the proposed architecture.

The proposed architecture consists of two parts and is visualized using the netron service^26^ in Fig. 3. First, an encoder from the MobileNetV2 architecture was taken.

Images with a width and height of 448 pixels are fed to the encoder input (model in Fig. 1). The output of the encoder is a tensor whose width and height are 28 pixels. Then, a decoder is added to this encoder, which consists of four upsampling layers (sequential, sequential_1, sequential_2, sequential_3), which increase the expansion to 448. Then comes the proposed activation function (CustomActivationLayer). This function takes the first channel of five that comes in and passes it through a sigmoid. In this process, it is necessary to apply a sigmoid function to the first channel of the output tensor. This transformation ensures that the resultant values lie within the range of 0–1. These values are subsequently interpreted as probabilities during the subsequent stages of post-processing. So, at the output of the neural network, we have the tensor $y_{{{\text{pred}}}} \in {\mathbb{R}}^{448 \times 448 \times 5}$.

Let's denote the first channel $h_{1}$. It represents the probability that pixel $\left( {x, y} \right)$ belongs to the center of the handle. The second channel $h_{2}$ represents the distance to the door handle. The remaining three channels $h_{3} , h_{4} , h_{5}$ are responsible for the normal of the door plane.

In this research, the function of the decoder is to allocate five specific values to each pixel within the image. The primary value is used for the localization of the handle within the image. The subsequent value facilitates the determination of this specific point in space relative to the camera. The remaining values are essential for ascertaining the orientation of the door handle, accommodating the variability in angles from which the camera may view the handle.

Thus, the decoder plays a crucial role in the architecture of the proposed neural network (see Fig. 3). Its main function is to recover the high-resolution original data from the low-dimensional representation generated by the encoder. In this architecture, the encoder compresses input images with a width and height of 448 pixels to a smaller tensor with a width and height of 28 pixels. This compression is achieved through a series of convolution layers that extract features from the input images.

After the encoder has created this compressed representation, the decoder samples this representation to the original 448-pixel resolution. This process involves four layers of sampling, each of which increases the spatial dimensions of the tensor, gradually expanding it to its original size. This reconstruction is necessary to preserve the spatial information and details lost during encoding.

In summary, the encoder extracts relevant features from the input images, compressing them into a lower-dimensional representation. The decoder then reconstructs this representation back to its original resolution, while also assigning specific values to each pixel to facilitate door handle localization, spatial positioning, and orientation determination. This interplay between the encoder and decoder enables the neural network to effectively analyze and interpret the input images in the context of door handle detection and localization.

### Postprocessing

The process of transmuting a depth image into a constellation of points necessitates the assignment of each pixel's depth value to a corresponding three-dimensional point within spatial coordinates. This intricate procedure mandates the acquisition of specific intrinsic camera parameters alongside the requisite depth data. Intrinsic parameters are typically constituted by the focal lengths ($f_{x}$ and $f_{y}$), the coordinates of the principal point ($c_{x}$ and $c_{y}$), and occasionally, parameters accounting for lens distortion.

A depth image is essentially an array of distance metrics, where each pixel details the measured distance from the camera to the point in the scene it denotes. In the endeavor to metamorphose a depth image into a point cloud, it is imperative to first procure the depth information. This involves the interpretation of each pixel within the depth image as a quantitative indicator of the range between the camera's lens and the scene's physical elements.

To calculate the 3D coordinates for each depth value at the pixel $\left( {x, y} \right)$, you can calculate the 3D point $\left( {x^{*} , y^{*} , z^{*} } \right)$ using the Eq. (1).

1 $$ \left\{ {\begin{array}{*{20}l} {{\text{x}}^{*} = \left( {\left( {{\text{x}} - {\text{c}}_{{\text{x}}} { }} \right) \cdot {\text{d}}} \right)/{\text{f}}_{{\text{x}}} ,} \hfill \\ {y^{*} = \left( {\left( {{\text{y}} - {\text{c}}_{{\text{y}}} { }} \right) \cdot {\text{d}}} \right)/{\text{f}}_{{\text{y}}} } \hfill \\ {z^{*} = d.} \hfill \\ \end{array} } \right., $$

where $f_{x}$ and $f_{y}$ are focal lengths, $c_{x}$ and $c_{y}$ are principal points.

The coordinates $\left( {x, y} \right)$ are determined at the locus where $h_{1}$ attains its maximal value. Consequentially, the distance to the handle is denoted as $\left( {h_{2} } \right)_{x,y}$, and the normal of the plane is represented by $q^{*} = \left( {\left( {h_{3} } \right)_{x,y} ,\left( {h_{4} } \right)_{x,y} ,\left( {h_{5} } \right)_{x,y} } \right)$. After this, a conversion is undertaken to translate these values into the parameters $p^{*}$ and $q^{*}$ (see Eq. 2).

2 $$ \left\{ {\begin{array}{*{20}l} {p^{*} = \left( {x^{*} ,y^{*} ,\left( {h_{2} } \right)_{x,y} } \right),} \hfill \\ {q^{*} = \left( {\left( {h_{3} } \right)_{x,y} ,\left( {h_{4} } \right)_{x,y} ,\left( {h_{5} } \right)_{x,y} } \right).} \hfill \\ \end{array} } \right. $$

### Loss function

At the output of the neural network, we have the tensor $y_{{{\text{pred}}}} \in {\mathbb{R}}^{448 \times 448 \times 5}$. For each training set, we have $y_{{{\text{true}}}} \in {\mathbb{R}}^{448 \times 448 \times 5}$. Then the Mean Squared Error (MSE) function looks like (3).

3 $$ {\text{MSE}}\left( {{\text{y}}_{{{\text{true}}}} ,{\text{y}}_{{{\text{pred}}}} } \right) = \frac{1}{448 \times 448 \times 5}\mathop \sum \limits_{{{\text{i}} = 1}}^{448} \mathop \sum \limits_{j = 1}^{448} \mathop \sum \limits_{k = 1}^{5} \left( {y_{{{\text{true}},ijk}} - y_{{{\text{pred}},ijk}} } \right)^{2} . $$

### Training setup

The training regimen was executed utilizing the TensorFlow framework. Training of the network was conducted on a singular GPU, utilizing the Adam optimization algorithm with a batch size of 32 instances. The learning rate was initially established at 0.01 and was methodically reduced to 0.001 across a span of 30 epochs. To facilitate the deployment of the trained models on mobile devices, a conversion process into the TensorFlow Lite (TFLite) format was implemented. This conversion is essential for ensuring that the models are compatible with the hardware and computational constraints inherent to mobile technology platforms.

### Augmentation

During the training phase, it is imperative to employ augmentation techniques that do not induce alterations to the intrinsic parameters of the camera. The intrinsic parameters encompass characteristics intrinsic to the camera that influence the projection of three-dimensional scenes onto two-dimensional images. These parameters entail focal length, coordinates of the image center, and distortion coefficients, among others. Maintaining the integrity of these parameters throughout the augmentation process signifies that any modifications must not simulate variations in camera focal length, position, or orientation. Adherence to this guideline is vital to ensure that the neural network is trained to recognize and accurately interpret the spatial positioning of objects, utilizing images that accurately represent actual scenes, as opposed to those in which fundamental perspective or geometric properties have been synthetically modified. Such a methodological approach is instrumental in circumventing erroneous learning scenarios, wherein the network may become unduly sensitive to non-realistic transformations in the visual appearance of objects or their spatial arrangement.

**Figure 4:**
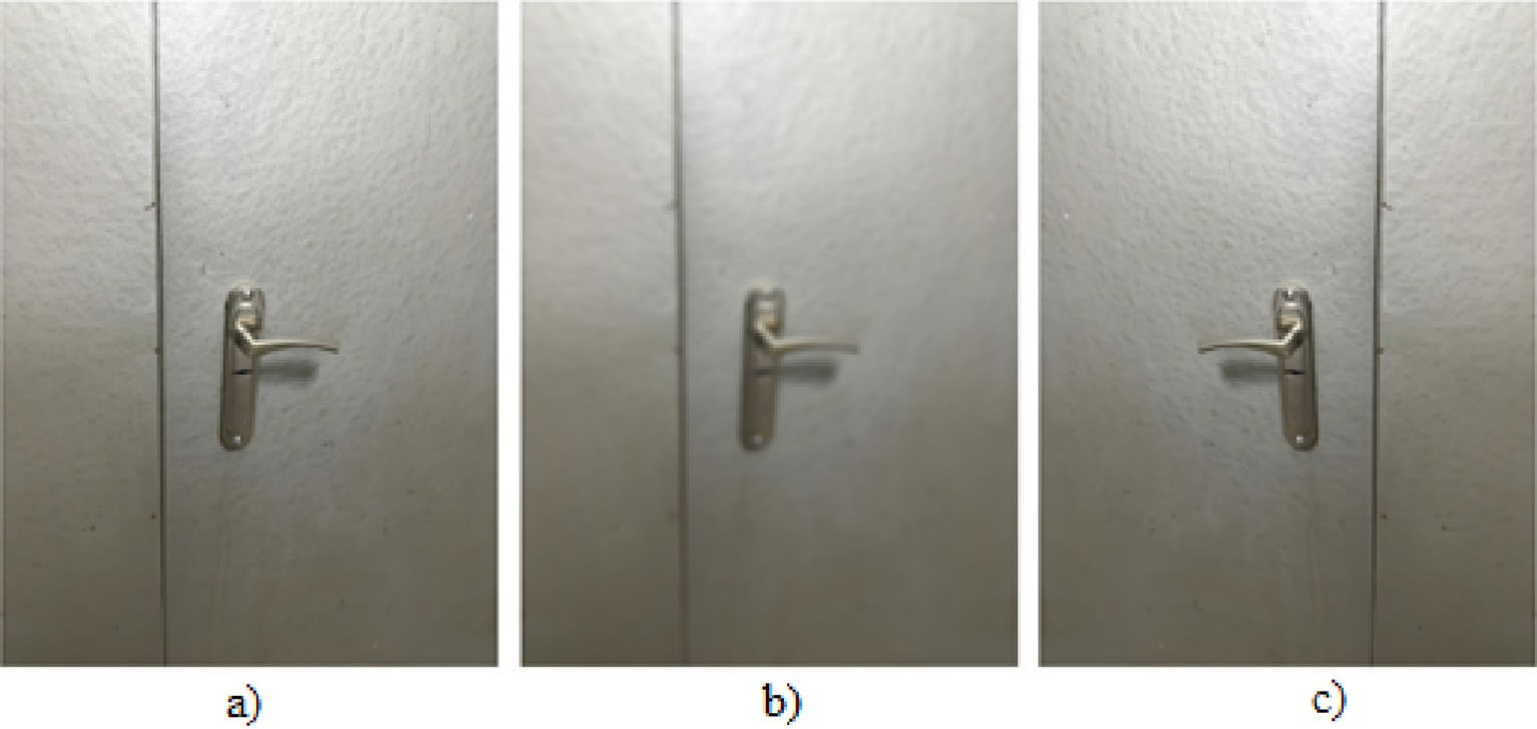
Data augmentation: (**a**) original, (**b**) blur, (**c**) flip.

In this research, two specific types of augmentation were implemented that respect the camera's internal parameters: blur and horizontal flip, as depicted in Fig. 4. These augmentation techniques have been identified as optimally congruent with the task at hand. The horizontal flip effectively mirrors the scenario of a door as viewed from the opposite side, while the application of blur can mimic the effect of slight camera defocus, both of which are practical phenomena that do not compromise the camera's intrinsic attributes.

### Algorithm using a trained neural network

As a result, the paper proposed an algorithm, the pseudocode of which is presented below (see Algorithm 1).

**Algorithm 1:**
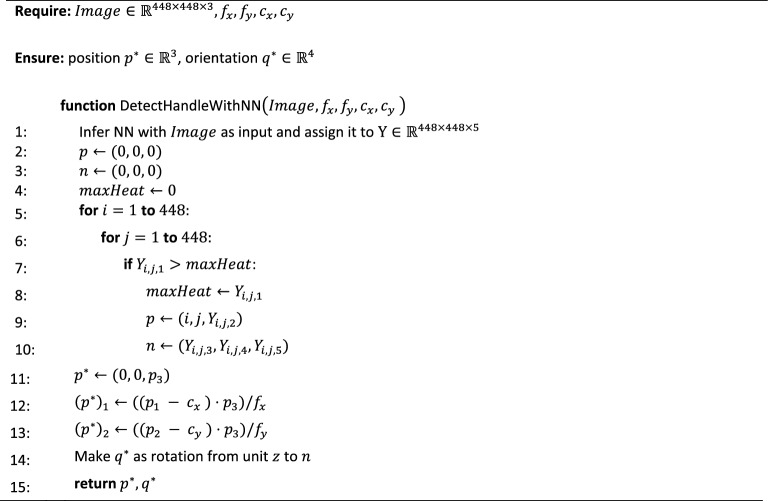
DetectHandleWithNN

Algorithm 1 delineates the comprehensive procedural pipeline which commences with the utilization of a neural network and extends to the post-processing phase that employs the intrinsic parameters of the camera. This systematic sequence culminates in the acquisition of the isometry of the doorknob, characterized by both a position vector and its corresponding quaternion.

## Results of numerical experiments

The TensorFlow computational framework served as the foundation for the development and training of the segmentation model^27^. This framework is extensively employed across sectors necessitating the analysis of voluminous datasets, such as computer vision, auditory recognition, pattern identification, and predictive modeling predicated on data. TensorFlow is compatible with both Central Processing Unit (CPU) and Graphics Processing Unit (GPU) based computations, and it boasts a malleable Application Programming Interface (API), facilitating its seamless incorporation into a diverse array of applications and platforms. The development of the program was undertaken utilizing the Linux Ubuntu 20.04 Long-Term Support (LTS) operating system^28^.

The model's training was performed using the Google Colaboratory service. This service proffers a virtualized environment that enables interaction with machine learning libraries, including TensorFlow, Keras, and PyTorch, among others, obviating the necessity for local environment configuration. A principal benefit of Google Colab is its provision of complimentary access to Graphics Processing Units (GPUs) and Tensor Processing Units (TPUs). This has rendered it a platform of choice among the research and development community for the training and prototyping of machine learning models.

For sensory data acquisition, the Intel RealSense D435 camera^29^ was employed, which stands as a sophisticated depth information acquisition device tailored for an array of applications. These applications span robotics, augmented reality, three-dimensional scanning, and various computer vision undertakings. As a constituent of the Intel RealSense suite of depth cameras, it leverages a synthesis of infrared sensors and camera technology to capture depth data. Moreover, the RealSense software package^30^ encompasses a calibration utility, enabling enhanced precision in the calibration process and the extraction of intrinsic camera parameters, inclusive of distortion coefficients.

### The result is on a specific door handle

**Figure 5:**
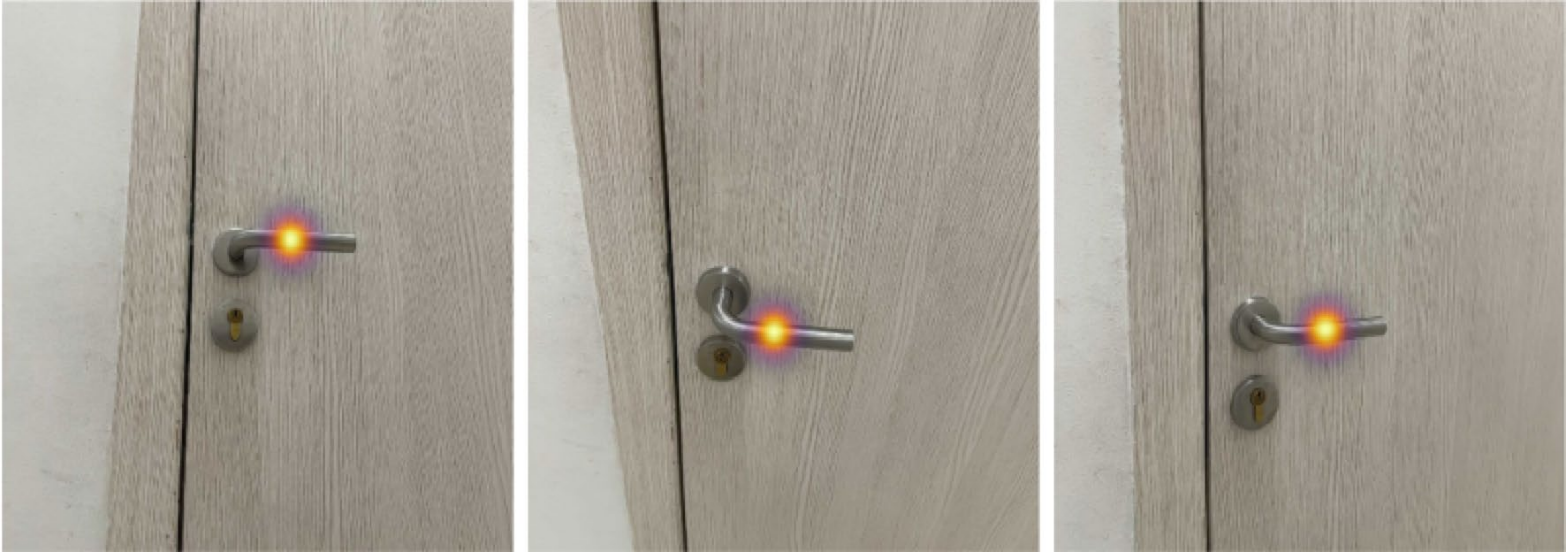
Visualization of the first channel, which is responsible for the probability distribution of the center of the door handle.

**Figure 6:**
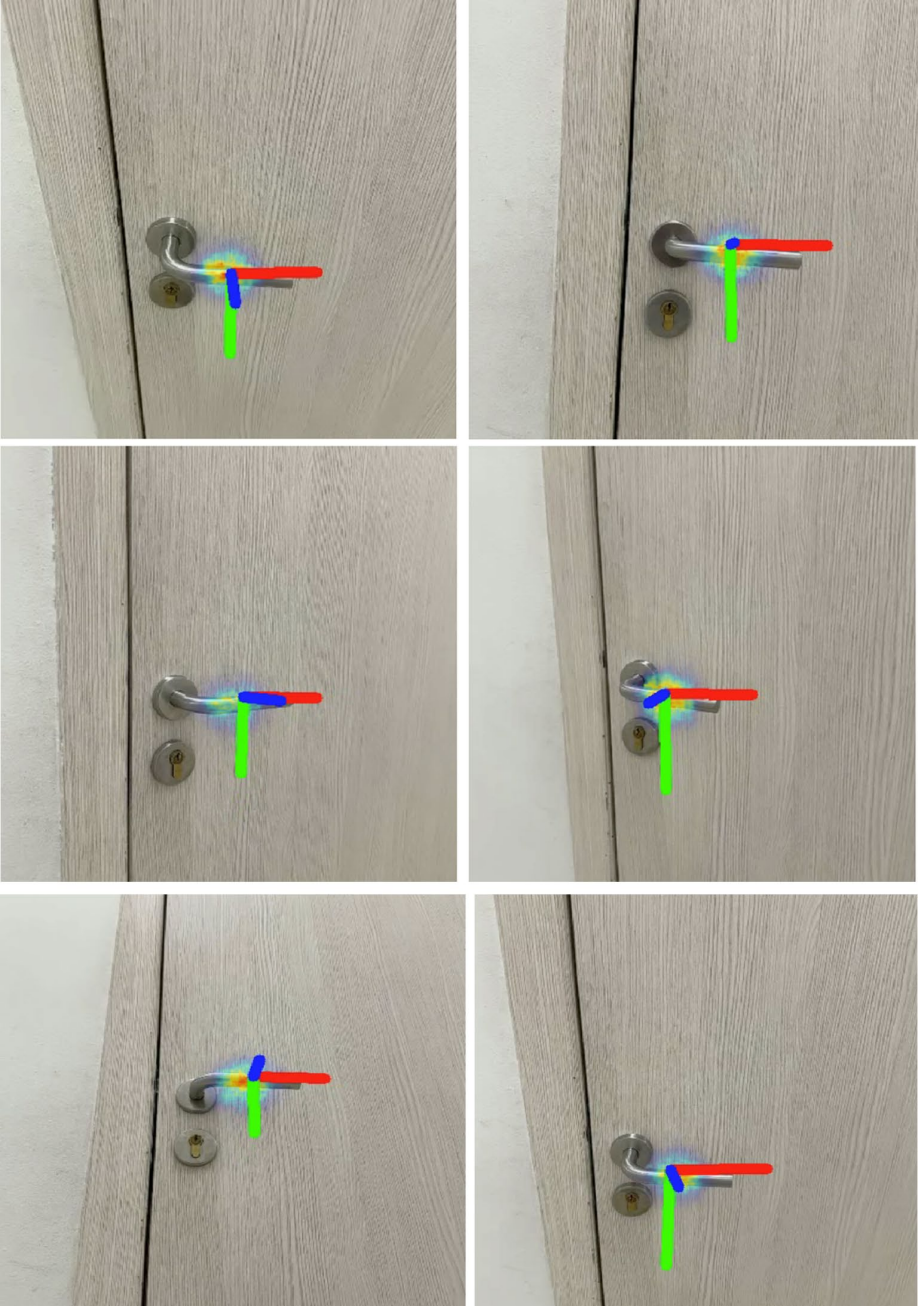
The results of the algorithm on the door handle from several angles.

In Fig. 5 visualizes the first channel, which is superimposed on the image. The model exhibits the capacity for real-time operation, achieving a processing throughput of 16 images per second. Illustrated in Fig. 6 is the algorithm's performance on a doorknob that was not encompassed within the training dataset. Inspection of this figure reveals that the model has adeptly adjusted and is capable of identifying the isometric parameters of the door handle with considerable precision.

Specifically, on this test door handle, the model has an error of 1.5 cm in position and a 5° angle of deviation from the door normal.

Additionally, the model underwent testing with four door handles that were not included in the training dataset, indicating that images of these handles from any perspective were absent from the dataset. The comparative analysis of accuracy reveals an error margin of 1.5 cm, suggesting that the model demonstrates commendable generalization capabilities, provided that a camera with identical intrinsic parameters, as used in the dataset, is employed for capturing each photo.

### Comparison with other detection models

**Figure 7:**
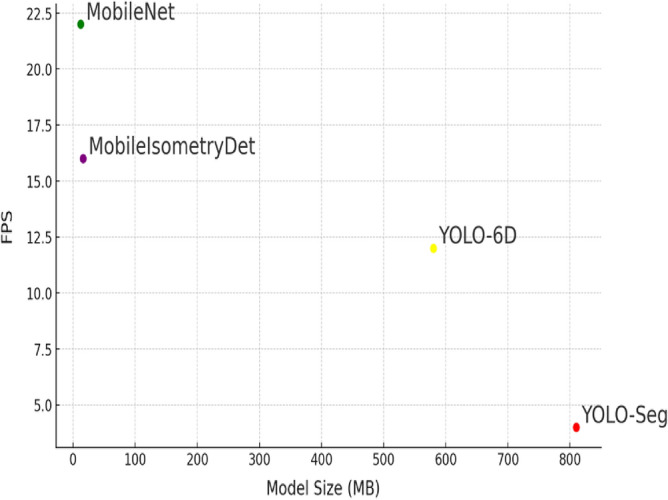
Comparing size and FPS with other models.

Quantitative indicators for comparing the efficiency of the proposed model with existing analogs are presented in Table 2. The proposed model has a fairly high FPS and is not large. It is capable of processing 16 frames per second and occupies 16 megabytes (see Fig. 7).

**Table 2:** Quantitative indicators for comparing the effectiveness of the proposed model with existing analogs: processing speed and memory size.

Models	Model size (MB)	Processing speed (FPS)
YOLO-Seg	810	2
YOLO-6D	580	12
MobileNet	16	22
MobileIsometryDet	16	16

Figure 7 presents a comparative analysis of the proposed MobileIsometryDet model with other prevalent models, such as MobileNet^26^, YOLO-6D^31^, and YOLO-Seg^32^. As can be discerned from the figure, the MobileIsometryDet model exhibits superior processing speed compared to the current benchmark in 3D object detection, YOLO-6D. Furthermore, the MobileIsometryDet model processes only six frames per second less than a model designed for a more rudimentary task of classification MobileNet. The proposed MobileIsometryDet model has a fairly high FPS and a small size. It is capable of processing 16 frames per second and takes up 16 megabytes. Consequently, this efficiency positions the MobileIsometryDet model as a viable option for deployment on mobile devices, an attribute that is particularly pertinent for the majority of autonomous mobile robots.

## Conclusions

This study marks a noteworthy advancement in the realm of algorithm development for the identification of door handle positions utilizing RGBD camera data. The research entailed a sequence of essential phases, commencing with the compilation and annotation of a substantial dataset comprising 5000 images, each annotated with the bounding box (BB) vertices' coordinates of door handles. This preliminary stage was pivotal in guaranteeing the effective training of the neural network.

The foundational element of this development was the MobileIsometryDet architecture, an innovative integration of an encoder based on MobileNetV2 and a decoder facilitating upsampling to a resolution of 448 pixels. A uniquely devised activation function was employed within this architecture, optimizing the efficiency of processing the network's output. Throughout the model training phase, the MSE function was utilized, enabling the precise quantification of discrepancies between predicted outcomes and actual results. This aspect is crucial in enhancing the accuracy of the model.

The study placed significant emphasis on data augmentation, particularly methods that do not alter the camera's intrinsic parameters. This approach ensured a superior quality of network training, equipping the model to adapt to diverse lighting scenarios and perspectives. The implementation of blur and horizontal flip as primary augmentation techniques contributed to increased variability in the training dataset, maintaining the data's relevance.

One of the paramount achievements of this work is the model's capability to operate in real-time, processing up to 16 images per second, indicative of the model's high efficiency and rapid processing ability. This feature is critically important for the practical application of the model. The algorithm demonstrated exceptional adaptability and precision in identifying isometric parameters of objects, even when tested on door handles not included in the training dataset.

The ability to recognize and interact with doorknobs signifies a significant advancement toward integrating robots seamlessly into everyday life, thereby improving human productivity and overall quality of life. This capability extends the potential applications of autonomous robots, making them more effective in diverse scenarios such as assisting individuals with disabilities, enhancing industrial automation, and contributing to household convenience.

In summation, this research exemplifies a successful approach to the intricate task of doorknob recognition through the application of deep learning techniques. Future investigations could explore the expansion of the dataset to encompass a wider array of doorknob types and lighting conditions, thereby enhancing the model's applicability across varied real-world scenarios.

Moreover, the integration of the developed model with other robotics systems, such as autonomous navigation or smart home technologies, presents a promising avenue for creating more sophisticated and functional solutions.

Continued research is imperative to mitigate the impact of external variables, such as fluctuating lighting conditions or visual obstacles, on the model's accuracy and stability, further refining the system's reliability and operational efficiency under varying usage conditions. Despite this, experiments conducted demonstrate the approach's notable reliability in detecting door handles, even in scenarios where they haven't been seen before, the material of the door handles changes, or the lighting conditions vary significantly. Leveraging data from an annotated set of 5000 images with corresponding coordinates of the door handle bounding box, the algorithm operates with RGBD images and internal camera parameters to accurately determine the handles' parameters. Future research plans include conducting additional experiments and further diversifying the test data.

Potential collision hazards arising from accuracy and uncertainty issues include unintended contact with objects or obstacles due to inaccurate object detection and unpredictable robot behavior. To address these challenges, countermeasures such as sensor redundancy, dynamic collision avoidance algorithms, safety margins, adaptive grasping strategies, and continuous monitoring can be implemented to enhance the robot's ability to perceive its environment accurately and react effectively to potential collision hazards.

## Data Availability

The datasets generated and analyzed in this study are not publicly available due to restrictions their containing information that could compromise the privacy of research participants, but can be obtained from the respective author upon reasonable request.
